# Variability in Echolocation Call Intensity in a Community of Horseshoe Bats: A Role for Resource Partitioning or Communication?

**DOI:** 10.1371/journal.pone.0012842

**Published:** 2010-09-17

**Authors:** Maike Schuchmann, Björn M. Siemers

**Affiliations:** Sensory Ecology Group, Max Planck Institute for Ornithology, Seewiesen, Germany; University of Pretoria, South Africa

## Abstract

**Background:**

Only recently data on bat echolocation call intensities is starting to accumulate. Yet, intensity is an ecologically crucial parameter, as it determines the extent of the bats' perceptual space and, specifically, prey detection distance. Interspecifically, we thus asked whether sympatric, congeneric bat species differ in call intensities and whether differences play a role for niche differentiation. Specifically, we investigated whether *R. mehelyi* that calls at a frequency clearly above what is predicted by allometry, compensates for frequency-dependent loss in detection distance by using elevated call intensity. Maximum echolocation call intensities might depend on body size or condition and thus be used as an honest signal of quality for intraspecific communication. We for the first time investigated whether a size-intensity relation is present in echolocating bats.

**Methodology/Principal Findings:**

We measured maximum call intensities and frequencies for all five European horseshoe bat species. Maximum intensity differed among species largely due to *R. euryale*. Furthermore, we found no compensation for frequency-dependent loss in detection distance in *R. mehelyi*. Intraspecifically, there is a negative correlation between forearm lengths and intensity in *R. euryale* and a trend for a negative correlation between body condition index and intensity in *R. ferrumequinum*. In *R. hipposideros*, females had 8 dB higher intensities than males. There were no correlations with body size or sex differences and intensity for the other species.

**Conclusions/Significance:**

Based on call intensity and frequency measurements, we estimated echolocation ranges for our study community. These suggest that intensity differences result in different prey detection distances and thus likely play some role for resource access.

It is interesting and at first glance counter-intuitive that, where a correlation was found, smaller bats called louder than large individuals. Such negative relationship between size or condition and vocal amplitude may indicate an as yet unknown physiological or sexual selection pressure.

## Introduction

Bats use echolocation to orient and to forage for flying insects in the dark. The distance at which a bat can detect airborne prey depends on the size and echo reflectance of the insect (the so-called target strengths), on the echo detection threshold of the bat's auditory system and, importantly, on the frequency and intensity of the echolocation calls [1], [2], [3], [4], [5], [6]. Lower call frequencies reach further than high frequencies, because they are less affected by atmospheric attenuation. The long, quasi constant frequency calls of large aerial hunting bats, when in the range between 10 and 20 kHz, might result in maximum detection distances of a few tens of meters for very large insects and for night-migrating passerine birds that some bats prey upon [3], [7]. But in most bats, prey detection ranges are restricted to at most a few metres [3], [5], [8], owing to the high absorption of ultrasound in air and the low target strength, especially of small insects.

Call frequencies of free flying bats in the field are well documented for many species [9], [10] and the influence of call frequency on detection range and size filtering, i.e., a perception bias toward large or small prey, has been studied in some detail [4], [11]. By contrast, call intensity received much less attention in classical field studies of bat echolocation (but see [12]). Donald Griffin, who discovered echolocation in bats and pioneered much of its scientific understanding, early on pointed out the ecological relevance of echolocation call intensities [13], but only recently a handful of studies started to accumulate knowledge on call intensity for free flying bats in the field [3], [5], [14], [15], [16]. They produced astonishing results such as source levels of 137 dB SPL – the intensity of a starting airplane – 10 cm in front of the bats' snouts [5]. Surlykke and Kalko [5] pointed out that sympatric bat species should differ in maximum detection ranges based on different call intensity. As echolocation call parameters are often shaped by ecological constraints related to habitat, and different echolocation call parameters result in differences in prey detection abilities, these differences, in turn, are thought to constitute a mechanism promoting resource partitioning among sympatric species via sensory specialization [5], [11], [17], [18].

In the present study, we measured maximum call intensities and corresponding call frequencies for the complete community of European horseshoe bats as a basis for calculating species- specific detection distances. Horseshoe bats lend themselves particularly well as a model system for studying the role of echolocation for resource partitioning. Indeed, the partitioning of frequency space used for echolocation has been investigated for several communities of horseshoe bats [17], [19], [20], [21], [22]. Call frequency scales with body size [17] and this likely explains the allocation of call frequency bands in horseshoe bat communities to some extent. There are, however, several cases where a species' call frequency conspicuously deviates from allometry [20], [21], [23], [24]. Both, ecological factors associated with partitioning of dietary resources [23] and a selection pressure for maintaining ‘private frequency bands’ for communication by echolocation (‘acoustic communication hypothesis’; [17], [21] have been proposed to explain these deviations from allometry. In support of the latter hypothesis, there is at least one documented case of likely acoustic character displacement for horseshoe bat call frequencies [20].

**Figure 1:**
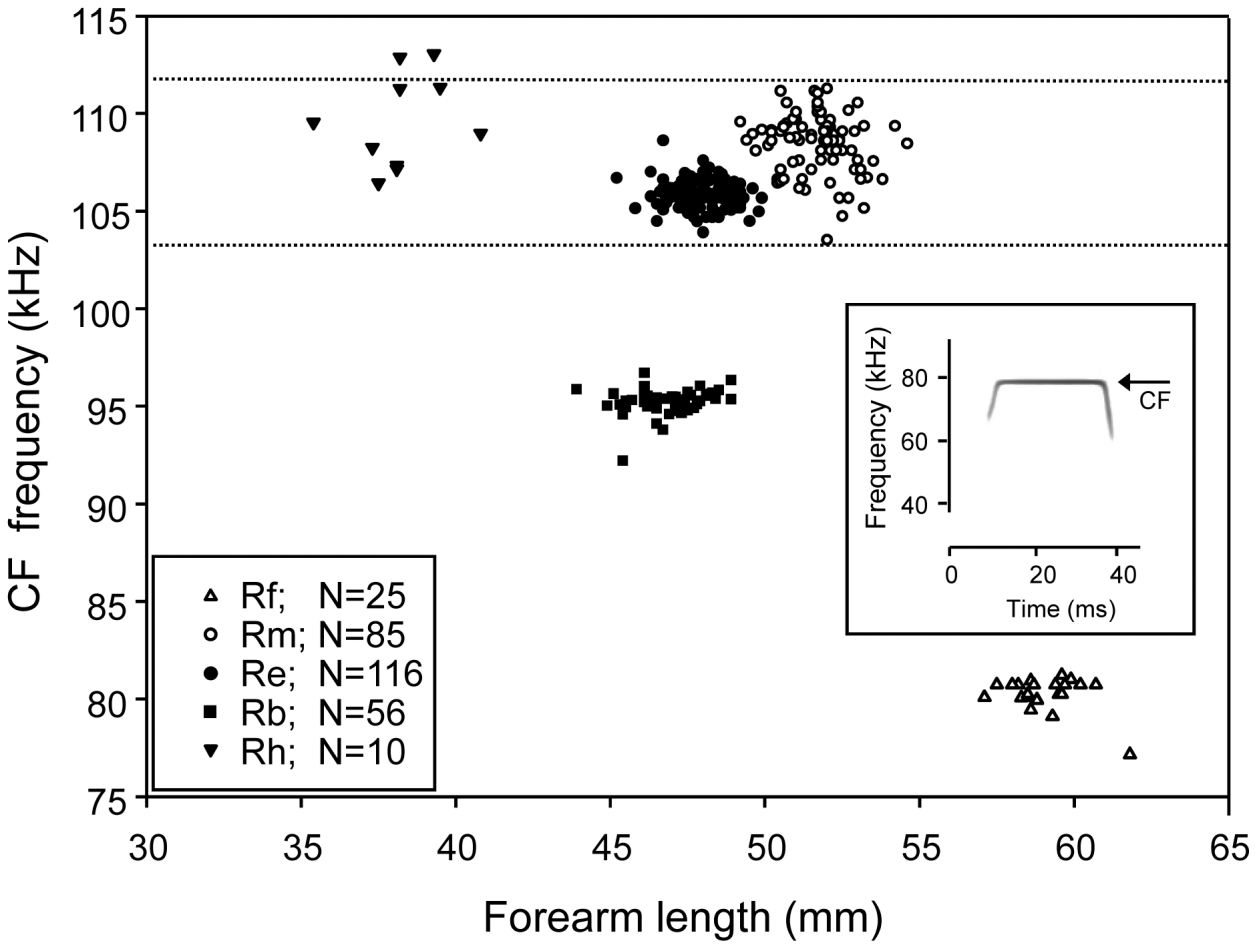
CF frequencies of sympatric horseshoe bats from Bulgaria are plotted against forearm length, the standard size measure in bats, for *Rhinolophus ferrumequinum* (Rf, N = 25), *R. blasii* (Rb, N = 56), *R. mehelyi* (Rm, N = 85), *R. euryale* (Re, N = 116) and *R. hipposideros* (Rh, N = 10). The peak echolocation call frequencies used by Rm strongly overlapped those of Re and Rh (dotted lines), while the bands used by Rf and Rb were clearly separated.

Four, and in some areas even five, species of horseshoe bat co-occur in Southeastern Europe, including Bulgaria, where we have conducted the present study. Interestingly, the call frequency bands of three species strongly overlap [22]. One of them, *Rhinolophus mehelyi*, uses a higher call frequency than predicted by allometry and its call frequency overlaps with that of the other two (compare Fig. 1). If it used the call frequency predicted by allometry, it would have a private frequency band on its own. While this contradicts predictions of the ‘acoustic communication hypothesis’, we have shown in a recent behavioral study that *R. mehelyi* individuals are able to discriminate conspecific echolocation calls from those of the partially overlapping species [24]. *R. euryale*, who's frequency band is completely encompassed within the broader *R. mehelyi* band (compare Fig. 1), showed a decreased ability of discriminating conspecifics calls from *R. mehelyi* calls, which lends some support to the ‘acoustic communication hypothesis’.

In the present study, we investigated whether *R. mehelyi* produces calls of especially high intensity and thereby compensates for the decrease in detection distance that results from the species' deviation from allometry to a higher call frequency.

There is limited evidence that call frequency encodes body size or sex and may hence function as an honest signal of quality for intraspecific communication [22], [25], [26], [27], [28]. A possible correlation of call intensity with body size or condition, which would allow echolocation to have a communication function [29], [30], [31], [32], [33], [34], [35], [36], has never been investigated in echolocating bats, and thus this is the first study to address this question.

The existence of acoustic communication signals in other groups of animals is supported by limited information on correlations between signal intensity, on the one hand, and body size or condition, on the other. For orthopteran insects, a positive correlation of body size and call intensity has been established [37], [38]. The same is true for toads [39], [40], [41] and for elephant seals [42]. In the American bison, *Bison bison*, a negative relationship between vocal amplitude and male quality has been found [43]. A negative correlation between body size and maximum song amplitude during interactive singing was also found for nightingales, while there was no correlation for two other songbird species [44].

In summary, our study aimed at answering the following questions:

1. Do call intensities differ among the five European species of horseshoe bat in an area of sympatry? And might call intensity differences play a role for niche differentiation?
2. Does *Rhinolophus mehelyi*, the species that calls “too high” for its body size, compensate for the detection range loss by using especially high call intensities?
3. Does maximum call intensity depend on body size or differ between sexes within species and thus might function as an honest signal for intraspecific communication?

## Results

### Overlap of frequency bands among species

In Bulgaria, where all five European horseshoe bat species roost in the same caves and forage partly syntopically (I. Dietz, C. Dietz, T. Ivanova & B.M. Siemers, unpublished data), only two of the five species use clearly separated CF- frequency bands (Fig. 1). CF frequencies used by *Rhinolophus mehelyi* (Rm) strongly overlapped with those used by *R. euryale* (Re) and *R. hipposideros* (Rh), while the CF frequencies used by *R. ferrumequinum* (Rf) and *R. blasii* (Rb) were clearly separated (Fig. 1). Statistically, the CF frequencies of the three overlapping species differed (One-way ANOVA, F_(2, 131)_, p<0.001; Bonferroni-corrected p-values<0.05 for all three pairwise post-hoc comparisons), but classification of individual CF by means of discriminant function analysis resulted in low levels of correct species assignment of Rm and Rh (Rm 35.4%, Rh 50.4%; chance level at 33.3%), while Re was well classified (97.4%).

### Interspecific call intensity relations

**Figure 2:**
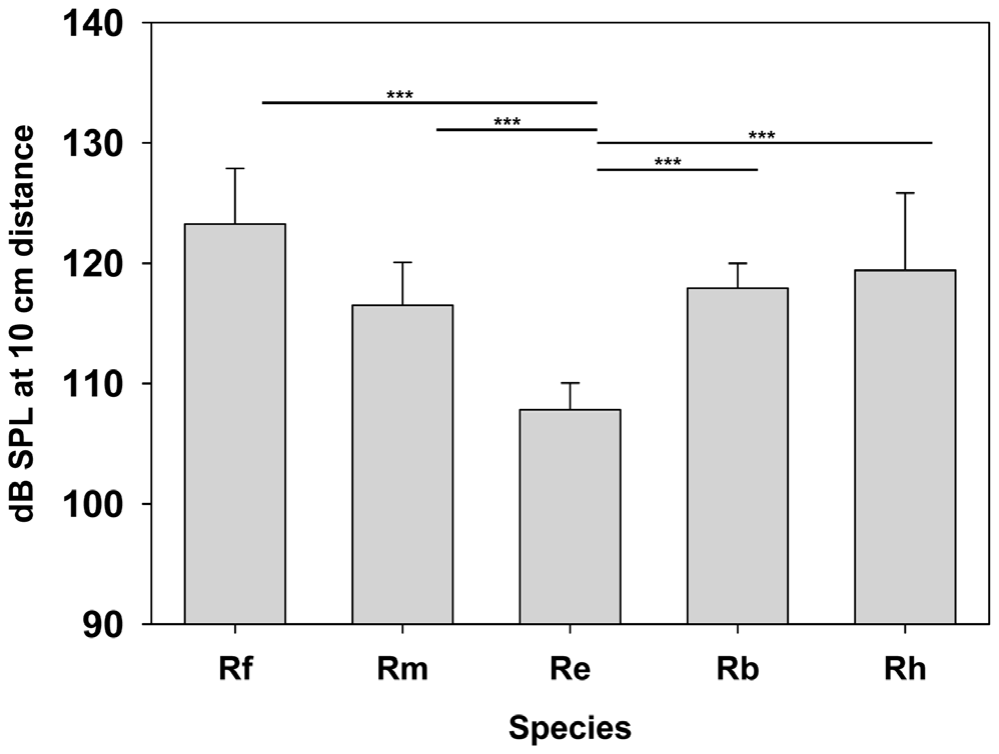
Interspecific call intensity relations. Maximum call intensities (in dB SPL; calculated for 10cm distance to the bats' nose) for *R. ferrumequinum* (Rf, N = 12), *R. blasii* (Rb, N = 6), *R. mehelyi* (Rm, N = 11), *R. euryale* (Re, N = 12) and *R. hipposideros* (Rh, N = 10). Lines and asterisks indicate the significant outcomes from all possible post-hoc pair wise comparisons (t-tests, p-values Bonferroni corrected, ***<0.0001).

Maximum call intensity differed considerably among the five sympatric horseshoe bat species (ANOVA, df = 4, p<0.001; for pair-wise post hoc comparisons, see Fig. 2). This difference was largely driven by Re, which produced call intensities 10 to 17 dB below the other species. Call intensities of the other four species, including Rm, the species that calls at a considerably higher CF than expected by allometry, did not differ significantly in the post hoc comparisons.

Call intensity was not correlated with forearm length across species, the standard measure for bat body size (Pearson correlation, r = 0.277, p = 0.652), and also not with body mass (Pearson correlation, r = 0.393, p = 0.512). There was also no correlation between call intensity and call frequency (r = −0.640, p = 0.244). Note that there is low statistical power for regression analysis, as our horseshoe bat community encompasses only 5 species.

### Intra-individual variation of call intensities and frequencies

**Figure 3:**
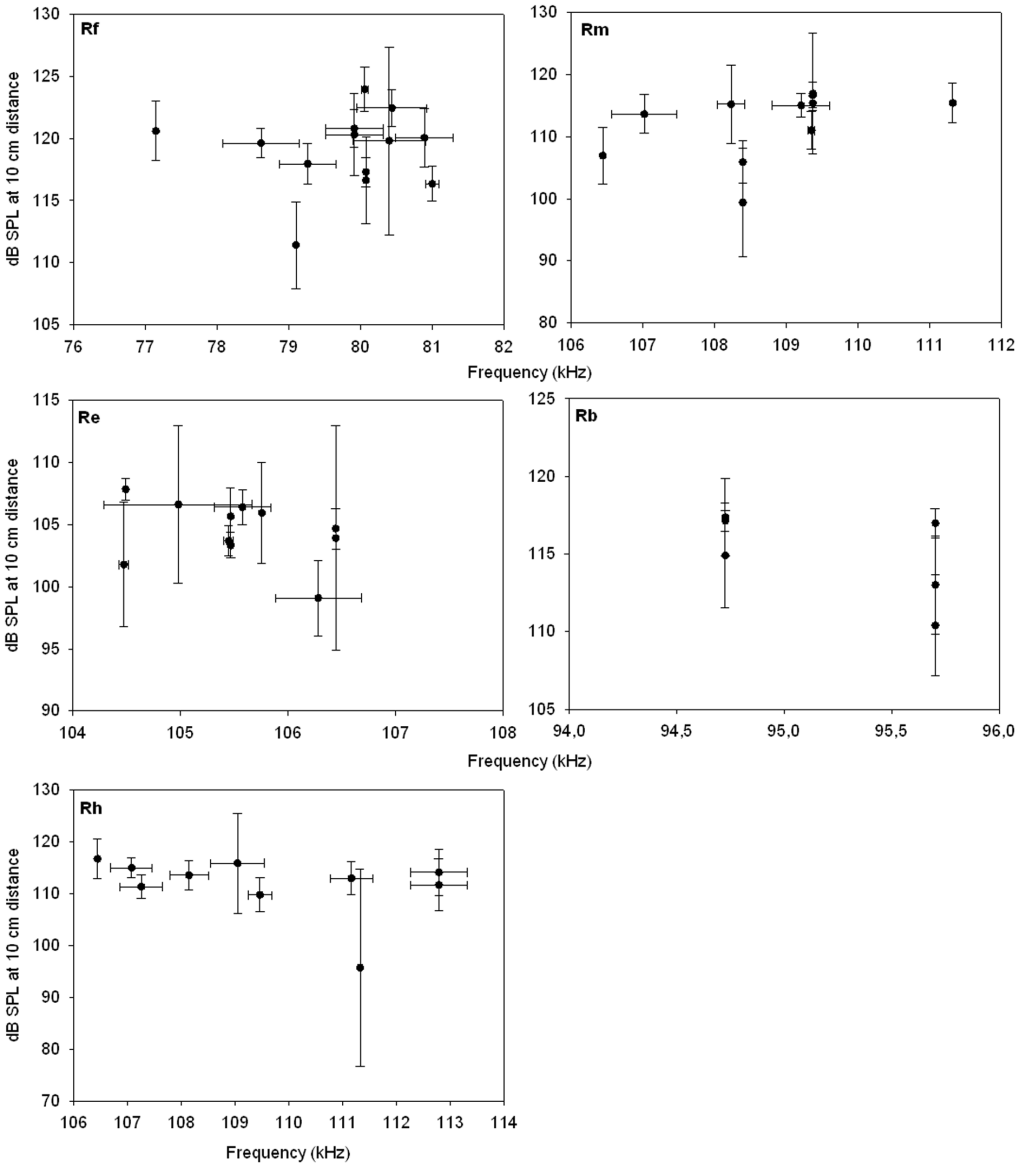
Intraspecific call intensity relations and intra-individual variation. Averaged call intensities of the six highest intensities of each individual (in dB SPL; calculated for 10cm distance to the bats' nose) for individual *R. ferrumequinum* (Rf, N = 12), *R. blasii* (Rb, N = 6), *R. mehelyi* (Rm, N = 11), *R. euryale* (Re, N = 12) and *R. hipposideros* (Rh, N = 10) are plotted against the corresponding averaged peak echolocation call frequencies. Error bars show the corresponding standard deviations.

While there was only little variation in intra-individual call frequencies within each species (species standard deviations ranged from 0 to 334 Hz), there was a larger variation of intra-individual maximum intensity per call sequence (species standard deviations ranged from 2.3 to 5.5 dB). Individual standard deviations are shown in Fig. 3.

### Intraspecific call intensity relations

Call intensity varied between individuals within the different species (maximum inter-individual intensity differences for Rb amounted to 6 dB; Re, 7.2 dB; Rf, 16.9 dB; Rh, 17.8 dB and Rm, 11.5 dB). The magnitude of the intraspecific call intensity range was not correlated with the number of tested animals (Pearson correlation, r = 0.448, p = 0.449).

**Figure 4:**
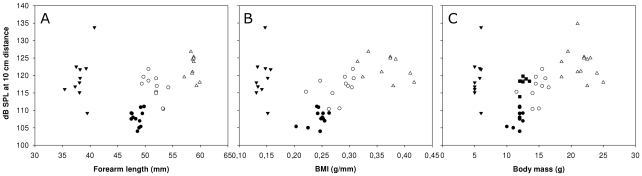
Intraspecific call intensity relations. Call intensities (in dB SPL; calculated for 10cm distance to the bats' nose) for individual *R. ferrumequinum* (Rf, N = 12), *R. mehelyi* (Rm, N = 11), *R. euryale* (Re, N = 12) and *R. hipposideros* (Rh, N = 10) are plotted against **A**: forearm length (FA), **B**: body mass index (BMI) and **C**: body mass. For statistics, see text. (Rb only used for Fig. 3 because of missing body size data)

Within species, there was no correlation between call intensity and CF frequency (Pearson correlation, all p>0.239, Fig. 3). We found a negative correlation between call intensity and forearm length (FA) for Rm (Pearson correlation, r = −0.646 p = 0.032, n = 11, Fig. 4 A, open circles). However, we could not confirm this relationship for the three other tested species (Rf, Re and Rh; Pearson correlation, p>0.215 Fig. 4 A). While we found a trend towards negative correlation between body condition (BMI) and call intensity for Rf (Pearson correlation, r = −0.576, p = 0.081, n = 10, Fig. 4 B, open triangles), this was absent in the other four species (p>0.451, Fig. 4 B). There was no correlation between body mass and call intensity for any of the species (Pearson correlation, all p>0.117, Fig. 4 C). Using the residuals from a regression of body mass on forearm length as an alternative measure for body condition showed the same results as for BMI.

Rh females had higher call intensities (p = 0.048; mean difference 8 dB) and also used on average 3 kHz higher call frequencies (t-test, p = 0.035) than Rh males. There were no sex differences in the body size parameters FA (t-test, p = 0.700) or BMI (t-test, p = 0.342) for Rh.

For Rf and Rm, we found no influences of sex on call intensity, frequency or body size (t- test; intensity: all p>0.277, frequency: p>0.415; FA: p>0.527). For Re and Rb, sex differences were not testable due to small sample size for one of the two sexes.

### Detection ranges

**Figure 5:**
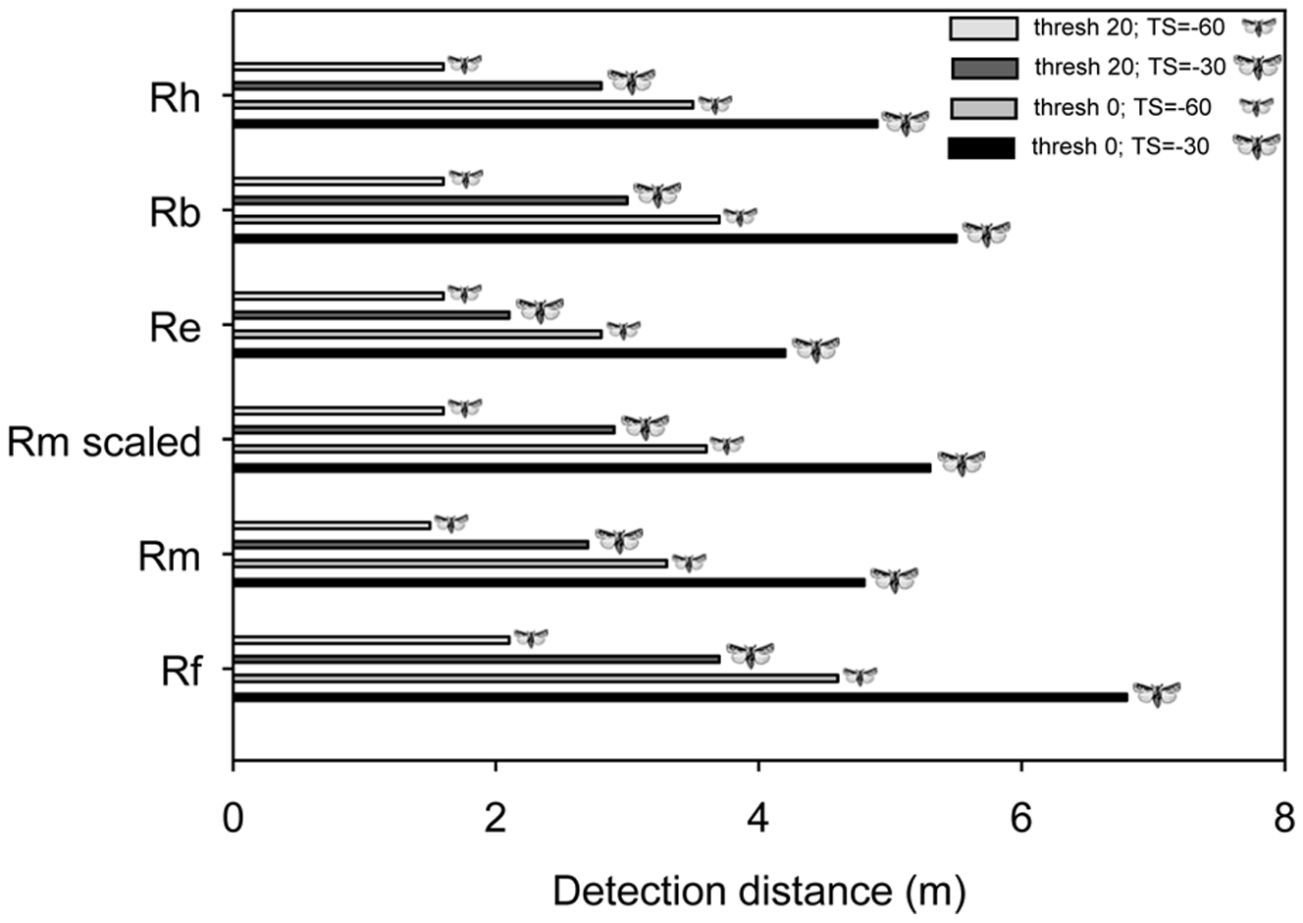
Estimated maximum prey detection distances for the five European species of horseshoe bat. The dark grey bars indicate maximum detection distances for large insects (target strength (TS) = −30 dB) for either an echo perception threshold (thresh) of 0 dB SPL or 20 dB SPL; the light grey bars show maximum detection distances for small insects (target strength (TS) = −60 dB) for either an echo perception threshold of 0 dB SPL or 20 dB SPL. Calculations were based on average maximum call intensities and an average peak echolocation call frequency as measured in this study. Species abbreviations as in the other figures. ‘Rm scaled’ indicated detection distances *R. mehelyi* would experience if the species called at the intensity we measured, but at a frequency as predicted by allometric scaling (97 kHz instead of 108 kHz).

Figure 5 shows species specific prey detection distances calculated for species-specific frequencies and species-specific maximum intensities measured in the present study (means). It includes estimates for two different target strengths (TS) and two different echo perception thresholds (see methods). Rf has the longest estimated detection range, followed by Rb, and Re the shortest. Rm and Rh have intermediate and very similar detection ranges for all conditions; yet, in one condition, Rh has a slightly longer detection distance (for DT = 0, Rh gains 10cm for TS = −60). If Rm would call at a frequency as predicted by allometric scaling (‘Rm-scaled’ at 97 kHz), detection distance would increase by maximally 50cm (DT = 0, TS = −30) in comparison with the species true CF (108 kHz).

## Discussion

### Interspecific call intensity relations

The current study shows that echolocation call intensities differed among the five species of the European horseshoe bat community. However, intensity differences were largely driven by one species, Re, calling at intensities 10–17 dB below the other four. All five horseshoe bat species had average maximum source levels between 107 and 123 dB SPL at 10cm distance from the bats' nose. These values fall within the lower part of the range of source levels measured in the field for free flying aerial insectivorous bats from the families Vespertilionidae, Emballonuridae, Mormoopidae and Molossidae (121–137 dB SPL [5], 110–115 dB SPL [12], 124–133 dB SPL [3], 133 [16], 121–125 dB SPL [14] all calculated for 10 cm distance to the bats' snout). For horseshoe bats (family Rhinolophidae), Waters and Jones [45] reported source levels for *R. hipposideros* flying in a room as 105 dB SPL and for a perched bat as maximum 100 dB SPL. We measured higher source levels for the same species, which was likely due to our more open recording situation. This comparison further substantiates our assumption that the values we measured are close to the maximum call intensities the species under study can produce.

In birds, larger species tend to produce vocalisations of higher intensity than smaller species [46]. A similar trend was found across eleven species of European vespertilionid bats by Holderied and von Helversen [3]. By contrast, we did not find a clear relationship between call intensity and body size for the five European horseshoe bat species. Interestingly, the by far smallest species, Rh (4–6 g) calls at nearly the same frequency and at the same intensity as the second largest species, Rm (12–16 g).

### Intensity adaption to avoid detection by tympanate prey?

In the current study, differences in call intensities were largely due to *R. euryale* calling at intensities 10–17 dB below the other four species. As there is evidence that the hearing sensitivity of insects is specific to the insectivorous bat assemblage that they are exposed to [47] and the diets of rhinolophids with peak frequencies >80 kHz often consists mainly of (tympanate) moths [48], it would be conceivable that selection may favour *R. euryale* calling at lower intensities to avoid detection by tympanate prey (i.e. driven by prey defences).

Nevertheless, in the current study system, moth hearing is a very unlikely explanation for why *R. euryale* calls at low intensities. First, it calls at around 105 kHz, i.e., way above the typical upper moths hearing threshold. Second, also *R. mehelyi* – similar frequency as *R. euryale*, but much higher intensity – eats mostly moths [49]. Even *R. ferrumequinum* that call at about 80 kHz and much louder than *R. euryale*, have a moth-dominated diet in our study area (I. Dietz, unpublished). Thus, for bats in Bulgaria that have call frequencies >80 kHz (and *R. euryale* has 105 kHz!), low call intensities do clearly not appear necessary for successful moth hunting.

### Niche partitioning through sensory specialisation?

As call intensity differences among the European horseshoe bat species were not clearly size-dependent, they might rather reflect adaptations to different foraging situations or habitats. But in bats body size influences foraging habitat as well so any difference in echo calls should also be reflected in body size. Several studies suggested that sensory specialisation, especially with relation to bat echolocation, might play a role for niche differentiation within animal communities [11], [18], [50], [51]. Specifically, differences in sensory performance can result in differential access to food and thus contribute to resource partitioning between potentially competing species [23], [52]. To estimate and compare prey detection abilities for the European horseshoe bat community, we calculated species-specific prey detection distances based on the call frequencies and intensities measured in this study (Fig. 5).

We found different detection distances among species. Rf will have the largest prey detection distance, followed by Rb. Rm and Rh will converge on very similar detection distances due to similarities in frequency and intensity of their echolocation calls and Re will have the shortest detection range due its considerable lower intensity. Note: Rm, the species that calls “too high” for its body size, does clearly not compensate for frequency-dependent loss in detection distance by using especially high call intensities. If Rm used a call frequency according to the genus trend (97 instead of 108 kHz), it would have a somewhat longer detection distance (see Fig. 5, ‘Rm scaled’).

The similar call frequencies and prey detection distances could enhance interspecific competition for resources between Rm and Rh. This might have been a driving factor for the evolution of divergent foraging habitat preferences, foraging behavior and wing morphology. Or else, these behavioral and morphological differences might enable stable coexistence of the two species, despite the extreme similarity of their echolocation systems. Rm prefers relatively open habitat [53] and regularly hunts from a perch [54], while Rh forages on the wing close to forests [55] and -especially in Bulgaria- around large trees within villages (I. Dietz; unpublished data, from: [56]). Furthermore, Rh has extremely short hand wings, enabling highly manoeuvrable search flight close to vegetation, while Rm has rather long hand wings [57] that allow fast and economic commuting flight, but come at an increased energetic cost for manoeuvring flights in vegetation [58]. While its is not possible to determine whether these behavioural, morphological and resulting ecological differences are the result of past competition or rather arose for different reasons, they in any case show that sensory separation is not mandatory for the coexistence of closely related species in a habitat-rich landscape.

Although Rm and Rh are likely able to detect similar sized prey at similar distances, they differ in prey processing abilities (Rh has a lower bite force than Rm and takes longer to chew up large prey; S. Greif, D. Schmieder and B.M. Siemers, unpublished) and also in the typical prey size used [56]. This might suggest, that the bats actively select their prey among the perceivable insects (comp.[59]).

### Can call intensity have a function for communication?

The intra-individual variability of the maximum call intensity for different call sequences averaged 2–5 dB per species (see Fig. 3), while variation in call frequency in perched bats was much smaller. Call frequency thus might be used as a reliably individual signature, while call intensity appears much less suited for efficient individual recognition. But does the intra-individual variation in call intensity question any potential suitability of call intensity for conveying communicative information? Below we argue that call intensity has a clear potential to convey quality-related information, despite that variability in call intensity as perceived by a listening bat will be still larger than the values we give. First, horseshoe bat calls are highly directional and thus a mismatch of the caller's head aim and the receiver's pinna directionality will result in a decreased perceived intensity. Additionally, the distance between caller and receiver will obviously greatly affect the intensity at the receiver's ear. It is, however, reasonable to assume that listening bats are able to reconstruct position [60] and head aim of the caller, at least for a perched bat, and thus will be able to reconstruct the emitted call intensity (i.e., the caller's source level). Second, echolocation calls serve primarily the purpose of echo generation for prey capture and orientation. Bats adjust emission level as a function of distance to prey [5] and as a function of general environmental echo reflectance (M. Schuchmann and B.M. Siemers, unpublished). However, also birds flexibly adjust their song amplitude to current environmental noise [61], [62], to the receiver distance [63] and other situational factors. Even in a standardized situation, individual song amplitudes can vary with interquartile ranges of 5 dB [44]. Despite this variability, bird song amplitude clearly has a communicative function [64], [65]. We thus argue that intra-individual variability in call intensity does not a priori preclude a potential function of echolocation call intensity for communicating size or quality. It will suffice that each bat utilizes its individual maximum amplitude at regular intervals.

Indeed, it took us only several seconds and maximally several minutes, to sample the individual maximum call intensity. Bats will easily be able to do the same, at least in a spatially defined situation with the caller hanging stationary in a roost or at a foraging perch. It is likely that bats can recognize at least some of their colony mates individually [66] and that they can do so from echolocation calls alone [32], independent of call source level. Bats spend their lives largely in dark or crepuscular environments and thus have not the possibility to visually assess body size and condition, competitive potential or putative mate quality of conspecifics. Echo-imaging will be of limited use here as well. We therefore see a strong potential for the idea that bats extract information on conspecifics' state or quality from their echolocation calls. Given they recognize the caller from intensity-independent call features [32], maximum intensity could be extracted and associated with the calling bat. Intensity-related information on individual state or quality could then not only used in immediate behavioural reactions, but also in future interactions with the calling bat.

### Intraspecific call intensity variation – a role for communicating size, condition or quality?

To our knowledge, our data set is the first to assess whether echolocation call intensity does indeed signals body size or condition and thus may play a role in intraspecific interactions. In contrast to our above expectations, we did not find a positive correlation between bat body size or condition and call intensity for any of four European horseshoe bat species. However, there was no correlation between body size and call intensity in Re and Rh. Where an effect was observable, the correlation between size and intensity was negative (Rm; a trend for Rf). Thus, within these species, smaller individuals used higher call amplitudes. This is somewhat counter-intuitive, as we had assumed that larger individuals should be capable of producing louder calls [67]. Indeed, a positive relationship between size and amplitude was established for several taxa [37], [38], [39], [40], [41]. Evidence from zebra finches suggest a direct fitness relevance of call amplitude, as females prefer louder over faint male song [65]. However, there are other examples, where a negative relationship between the amplitude of acoustic signals and body size or quality has been reported [43], [44].

Small or low quality animals might be ready to invest more into producing loud calls to try and make up for their inferior state. If they indeed can, this would question the honesty of the signal, however. In the case of echolocating bats, small or low condition individuals might increase foraging efficiency by using higher echolocation call intensities than conspecifics in better condition. Again, the question arises whether the measured “maximum” intensities are physiologically limited or rather under motivational control of the animal. The negative relation between size or condition and call intensity in bats, birds [44] and bisons [43] may also indicate an as yet unknown physiological or sexual selection pressure in need of further investigation.

While there is some evidence for sex-related differences in call frequency and temporal patterning in bats (e.g., [30], [68], [69], [70], [71] and other mammals e.g., [72], nothing is known about sex-related intensity differences in bat calls. This study for the first time shows sex differences in echolocation call intensities for bats. Specifically, we found that Rh females had on average 8 dB higher intensities than males. As far as sample sizes allowed testing, there was no body size relation or sex differences in call intensity for the other species. Whether the higher call intensities in Rh females play a role for communication or serve to (over) compensate the range loss resulting from the higher female call frequency (compare [70]) clearly deserves further study.

To further assess the importance of echolocation call intensity for communication, future studies will be necessary to test whether bats indeed use intensity differences among individual as a basis for decision-making and specifically tailor behavioral responses (such as avoidance/ approaching/ following behavior; changes in echolocation activity or in social calls; attention; courting etc.) as a function of other bats' call amplitudes.

## Materials and Methods

### Study area

This study was conducted at the Tabachka field station of the Sensory Ecology Group (MPI Seewiesen) that is run in cooperation with the Directorate of the Rusenski Lom Nature Park in the district of Ruse, northern Bulgaria. Four horseshoe bat species (Rm, Re, Rf and Rh) occur sympatrically in this area and roost in the same caves. A fifth species (Rb) only occurs more to the South, in the Eastern Rhodopes, where it is sympatric with the other four European horseshoe bat species. We captured Rm, Re, Rf and Rh for sound recordings from May to September 2007 and 2008 at four different caves close to the field station and Rb at one cave in the Eastern Rhodopes in 2007.

### Animals, capture and husbandry

Call intensity and frequency were measured from six Rb (all females), 12 Rf (8 females, 4 males), 12 Re (1 female, 11 males), 11 Rm (3 females, 8 males) and 10 Rh (4 females, 6 males) in 2007 as described below. To enlarge our sample size for the analysis of frequency band overlap, we used call frequency data from additional recordings from 2008 (13 Rf, 41 Re and 38 Rm) and data from Siemers [22]; 50 Rb, 63 Re and 36 Rm; recorded in 2001). These additional data were all from bats captured at the above mentioned caves. We only used adult bats. Bats were permanently marked by rings or transiently by wing punches or fur marks to avoid measuring any individual twice. The likelihood of an inadverted recapture was anyway minimal, as the colonies consist of several thousand bats.

Bats were sexed by inspecting the genitalia, weighed (Pesola lightline 10050; precision 0.5 g and Pesola lightline 10020; precision 0.2 g) and forearm length was taken (dialmax precision caliper; precision 0.1 mm). Forearm measurements were available for 50 Rb, 116 Re, 22 Rf, 85 Rm and 10 Rh. For these individuals, we calculated individual body mass indices (BMI) as BMI (g/mm) = mass/forearm length [73]. For mass, we always refer to capture weight.

Bats were captured at or close to the caves with a harp trap, mist nets or hand-nets. Captured bats were kept for a maximum of 5 days in a holding room at our field station (temperature around 25°C, humidity around 75%; close to natural conditions in the caves, own data). Light was turned off at dusk and was turned on at dawn. Bats were housed in screened tents (Tatonka, single moskito dome; 220×90×110 cm) with free access to water. Call recordings always occurred in the first or latest second night. Bats were fed mealworms between sound recordings to keep them motivated and received food (moths, mealworms) *ad lib* after the experiments. Capture, husbandry and behavioral studies were carried out under license of the responsible Bulgarian authorities (MOEW-Sofia and RIOSV-Ruse, 57/18.04.2006 and 100/04.07.2007).

### Experimental setup

When approaching echo targets, bats adjust their call intensity to keep echo levels constant [74]. It is thus likely that they adapt emission levels to the echo reflection properties of the environment to some degree; fainter in confined, echo-cluttered environments and louder in more open, less cluttered situations. As we were interested to assess high – ideally maximum – intensity calls, we recorded the bats in a large room (8×4×2.5 m) with sound attenuating material (felt-like insulating material ‘Velter’, thickness 5mm, Arbanasy EOOD, Veliko Ternovo, Bulgaria) on the walls. To mimic a perch hunting situation, a typical foraging style of many horseshoe bats [68], [75], we trained the bats to perch on a wooden basket, which was mounted on the shorter wall with maximum distance to each side wall, floor and ceiling. Given the high call frequencies of the bats (80–115 kHz, see Fig. 1) the resulting strong attenuation of calls and echoes with distance and thus the restricted perceptual space of horseshoe bats, 8 m clutter-free space ahead was certainly a very open situation for the bats. This assumption was corroborated by the fact that the bats produced consistently and considerably louder calls in the large room than in more confined recording environments (Schuchmann and Siemers, unpublished data).

The bats' sonar emissions were picked up with a ¼ inch measurement microphone (Type 40 BF, GRAS, Denmark) that was installed exactly 1 m in front of the bats' head. The microphone was mounted on a preamplifier (Type 26AA, GRAS, Denmark). These components were connected to an ultrasound recording interface (UltraSoundGate 416H, Avisoft Bioacoustics, Berlin, Germany), which was plugged in a lap top (IBM Lenovo ThinkPad). Calls were recorded via Avisoft recording software (Avisoft Recorder USGH, Avisoft Bioacoustics, Berlin, Germany) with a sampling rate of either 250 kHz or 500 kHz with 16 bit depths. The microphone was calibrated before the start of the recordings with signals of known intensity. Within the frequency range from 20 kHz to 140 kHz, the frequency responses of all recording components were flat (+/−3dB). Recording only took place when the bat directly called in direction of the microphone. This was monitored by the experimenter who was positioned close to the perched bat.

### Determination of call frequencies

The CF frequency of echolocation calls in horseshoe bats is a narrowband part of the call where the call stays constant at one frequency for around 90% of call duration (see inset in Fig. 1). To define the CF frequency we determined the temporal midpoint of the constant-frequent part of the call and analysed a time window of 10 ms around it. We scored the highest frequency in this 10 ms window as the CF frequency of this call. For all 2007 and 2008 recordings, the CF frequency of the second harmonic was read from a 512 points FFT (Hanning window; frequency resolution 0.5 kHz for 250 kHz sampling rate and 1 kHz for 500 kHz sampling rate) using a custom Matlab (Version 7.4, The MathWorks, Germany) routine or the colour spectrogram software Selena (University of Tübingen). For the 2001 recordings, frequency resolution for the CF frequency was 7.5 Hz (for details see Siemers et al [22]). In all cases, frequency resolution was fine enough to delimit species' frequency bands, as these typically span several kilohertz [17], [19], [20], [21], [22].

### Determination of call intensities

Echolocation call recordings were analyzed in Matlab 7.4 (Mathworks). We used the script ‘Callviewer’ (written by Mark Skowronski; see Skowronski and Fenton [76]) and a self-written Matlab routine to determine maximum call intensity for each bat. First, all recordings were cut in 20 s pieces to ease processing. All resulting pieces from each bat were automatically scanned for echolocation calls and the call with the highest intensity in the CF-part of the second harmonic was determined via Callviewer. To these calls, a FIR bandpass filter (+/−5% of CF-frequency of the call) with an order of 128 was applied. Next, we determined the temporal midpoint of the call and calculated 512-point FFTs with no overlap on the central 10 ms of the call. For each FFT-block, the frequency bin with the maximum value was determined. We took the average of all maximum bin values in the 10 ms window as our measure of maximum call intensity for each call sequence. For each bat, we determined the loudest call over all sequences available and scored it as maximum call intensity of that bat.

We recorded and analysed around 1500 calls per bat (range 134 to 4044); there was no relationship between number of analyzed calls and maximal call intensity (linear regression analysis, all p-values>0.07). The intensities are given in dB pe SPL re: 20 µPa [77] for a reference distance of 10 cm from the bat's nose, i.e. the value that would have been measured at a distance of 10 cm.

To get a measure of intra-individuals variation in call intensity we selected the six call sequences with the highest intensity calls from all call sequences per animal. We computed the average and the standard deviation for these six highest intensities and also for the corresponding call frequencies to determine intra-individual variation of intensity and frequency.

### Maximum detection distance

We estimated the maximum detection distances for two different insect sized targets for each of the five horseshoe bat species as a function of their echolocation call frequency and their maximum echolocation call intensity. Building on Mohl's [6] sonar equation, we calculated the sound pressure level of a returning echo as *E = SL+TLS+TLA+TS*. Here, SL is the emission level in dB SPL. TLS is the transmission loss owing to spherical spreading as a function of distance both on the way from the bat to the prey and back: 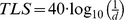. TLA is the transmission loss owing to absorption and was calculated for species-specific echolocation call frequencies (species means as determined in this study; Rf = 80 kHz, Rm = 108 kHz, Re = 106 kHz, Rb = 95 kHz, Rh = 110 kHz) and the same temperature (24°C), air pressure (101, 325 Pa) and humidity (65%) as measured in the flight room during the call intensity measurements: 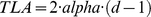. For the calculation of *alpha*, which is a function of call frequency (*f*), airpressure (*p*), temperature (*T*) and relative humidity (*r*), we followed the standard formula provided, e.g., by Stilz [78]. The target strength TS is defined as the logarithmic ratio of incident acoustic energy to the reflected energy, measured at a certain distance from the target along its acoustic axis [6]. Our reference distance was defined as 1 m. We considered two different types of targets: a small prey with a TS of −60dB (e.g. small moths or dipterans) and a larger prey with a TS of −30dB (e.g. large noctuid moths [2], [4]). Whether a returning echo still is detectable by the bat depends on the echo perception threshold, for which we assumed two different values; (1) 0 dB SPL, which is close to the standard mammalian hearing threshold under quiet conditions and is assumed by some authors also as echo detection threshold [3], [5], [8], [79], [80] and (2) 20 dB SPL, which represents a rather conservative estimate [5], [11], [81].
